# The surprising power of a click requirement: How click requirements and warnings affect users’ willingness to disclose personal information

**DOI:** 10.1371/journal.pone.0263097

**Published:** 2022-02-18

**Authors:** Robert Epstein, Vanessa R. Zankich

**Affiliations:** American Institute for Behavioral Research and Technology, Vista, California, United States of America; Universita degli Studi di Milano, ITALY

## Abstract

What kinds of information and alerts might cause internet users to be more cautious about what they reveal online? We used a 25-item survey to determine whether the strength of Terms of Service (TOS) warnings and the inclusion of a click requirement affect people’s willingness to admit to engaging in inappropriate behaviors. A racially and ethnically diverse group of 1,500 people participated in the study; 98.3% were from the US and India and the remainder from 18 other countries. Participants were randomly assigned to five different groups in which warnings and click requirements varied. In the control condition, no warning was provided. In the four experimental groups, two factors were varied in a 2 × 2 factorial design: strength of warning and click requirement. We found that strong warnings were more effective than weak warnings in decreasing personal disclosures and that click requirements added to the deterrent power of both strong and weak warnings. We also found that a commonly used TOS warning has no impact on disclosures. Participants in the control group provided 32.8% more information than participants in the two click requirement groups combined and 24.3% more information than participants in the four experimental groups combined. The pattern according to which people dropped out of the five different groups sheds further light on the surprising power of the click requirement, as well as on the importance of tracking attrition in online studies.

## 1. Introduction

Companies and governments are now collecting vast amounts of personal information online every day, and more people are becoming aware of how extensively they are being monitored. Relatively few people, however, are aware of the range of ways in which their private information is being used [1]. Some US states require immediate warnings when telephone conversations are monitored or recorded, presumably to give callers the option of moderating their speech, and research on cigarette warning labels suggests that salient warnings help some consumers behave more prudently [2]. What kinds of privacy-related warnings might cause internet users to be more cautious about what they reveal online?

People are becoming increasingly concerned about surveillance driven by new technologies. The National Security Agency (NSA) is a US intelligence and defense agency that specializes in cryptology and information assurance [3]. In 2013, whistleblower Edward Snowden alerted the American public about the NSA’s pervasive surveillance of US citizens, a move that resulted in an increase in disapproval of government surveillance, heightened concerns about technology use, and a reduction in visits to websites that government agencies might be monitoring closely [4, 5]. A year after Snowden’s disclosures, the Pew Research Center found that only 9% of American adults reported thinking that they have a high degree of control over how their data are being used, and only 6% reported confidence in the privacy and security of their data [1, 6, 7].

Most parents are also concerned about their children’s online behavior [8], and 61% report worrying that their teens are disclosing too much personal information online [9]. Meanwhile, teens are now sharing more information about themselves online than they have in the past, with only 9% of teen social media users reporting being “very concerned” about third-party access to their data [10]. In addition, adolescents’ concerns about online privacy are not associated with actual willingness to disclose, meaning that those who do express privacy concerns are not necessarily engaging in more privacy-protective behavior [11].

## 2. Privacy protections, threats, and behavior

### 2.1 Privacy protections outside the tech industry

Government often steps in to protect consumer data and soothe privacy concerns. Some US states require “dual consent” in phone calls, meaning that all participants on a call must be fully aware that the call is being monitored or recorded [12]. The US Federal Trade Commission (FTC) requires that any information provided by businesses that might affect consumers’ behavior must be accurate [13]. There also exist laws in the US–so-called “Peeping Tom Laws”–that make it a misdemeanor to spy on or photograph someone in a private place without that person’s consent [14]. These laws also prohibit nonconsensual video surveillance, or “video voyeurism,” in places where there is a reasonable expectation of privacy (e.g., bathrooms, bedrooms, changing rooms, etc.).

The US Privacy Act of 1974 protects records collected by the US government that contain citizens’ personal identifiers, including names and social security numbers [15]. The Privacy Act also states that individuals have the right to seek access to and request correction of any such records about them and prohibits collection and disclosure of such records without the consent of the individual to whom the records pertain. The US Fair Credit Reporting Act of 1970 holds credit reporting businesses responsible for the accuracy and security of personal information that is collected about consumers and then shared with third parties [16]. The US Gramm-Leach-Bliley Act of 1999 protects financial nonpublic personal information by requiring financial institutions to clearly and conspicuously explain their privacy practices and to safeguard any sensitive data they possess [17].

Healthcare records in the US are protected by the Health Insurance Portability and Accountability Act of 1996 (HIPAA) and other privacy laws that require healthcare providers to acquire patients’ written consent before disclosing their sensitive health information to other people and organizations [18, 19].

### 2.2 Privacy constraints on tech companies

The US Children’s Online Privacy Protection Act (COPPA) of 1998 was intended to protect the personal information of children 12 and under by prohibiting online companies from asking for any of their personally identifiable information without parental consent [20].

But technology companies that have emerged worldwide over the past two decades are largely unregulated, and it is only recently that a few aggressive laws and regulations have been implemented that attempt to safeguard consumer privacy. The most ambitious law passed so far is the European Union’s General Data Protection Regulation (GDPR), which became effective in 2018. Among other things, the GDPR guarantees, at least in theory, that consumers can have their personal data erased, can find out how their personal data are being used, and can shift their data to other platforms [21]. As a practical matter, however, it is not clear that the GDPR has actually changed pervasive business practices or has benefitted consumers, and some evidence suggests that because of the regulatory burden the GDPR presents, it has hurt small companies and startups in Europe while benefitting the Big Tech companies [22]. Meanwhile, several countries outside the EU have implemented similar regulations, and so has the US state of California [23]. More limited data privacy laws have been enacted by the US states of Nevada and Maine [24, 25].

Unfortunately, most if not all of the new and upcoming privacy rules give tech companies free rein when they have the consent of users, and users often have no idea they have given such consent [26–28]. Few users have ever fully read a Terms of Service (TOS) agreement or Privacy Policy, and tech companies often find ways around the rules [29–32]. “When you use our services,” begins Google’s 3,000-word Privacy Policy, “you’re trusting us with your information” [33]. A link to that Privacy Policy is embedded in Google’s 1,900-word Terms of Service Agreement [34]. Unfortunately, people are agreeing to the terms of both of these agreements even if they don’t know they are using a Google service, which is the case most of the time. Millions of websites incorporate Google Analytics, for example, which helps website owners track visitors to their sites [35]. But Google Analytics is invisible to users. Its presence on a website, however, allows Google to track everything users do on that website. Users have inadvertently given their consent when they have unknowingly started using a Google service, and that makes the GDPR and similar regulations largely ineffectual.

### 2.3 Other tech threats to privacy

Implied consent is just one of many privacy problems that new technologies pose. Because fortunes can be made quickly with newly deployed computer code, most new code is poorly written, which often means it is vulnerable to hacking and infiltration [36]. This puts users’ sensitive information, including login credentials, healthcare records, financial records, email content, and browsing history, at risk. Between March 2016 and March 2017, 1.9 billion usernames and passwords were exposed by data breaches and traded on black-market forums [37]. In 2010, it was discovered that Google Street View vehicles weren’t just taking pictures of people’s homes and businesses; they were also vacuuming up gigabytes of unprotected Wi-Fi data, including passwords, and they had been doing so in 30 countries for 3 years [38]. The ease of hacking, along with the fact that it is virtually impossible to erase data from the internet (all of which is vulnerable to hacking) [39], reminds us that the internet was not designed with security in mind.

Privacy is also at risk online because of toothless regulations and laws. COPPA, for example, supposedly shields children ages 12 and under, but a child of any age can gain full access to a pornography website simply by clicking a button reading “I am over 18.” One survey found that 7.5 million of Facebook’s users were under age 13, demonstrating how difficult it can be for sites to verify the ages of their users [40, 41]. COPPA also fails to protect young people over age 12, leaving a large gap in the protection of America’s youth.

Because corporations are driven by profit, their privacy policies tend to undermine user privacy rather than protect it, and they often use design features–or “dark patterns” [42]–to frustrate, confuse, or coerce users into participation [43–45]–a practice called “malicious interface design” [46]. Privacy policies are not only excessively lengthy (the average American would need to set aside almost 250 hours to properly read all the digital contracts they accept while using online services), they are also often written in language that is difficult to understand [47–49]. This is the case even for policies regarding sensitive health information [50].

### 2.4 Privacy-protective behavior

One would think that people concerned about privacy would make an effort to protect it, but this is often not the case. The gap between the concern people express about privacy and their actual privacy-protective behavior is called “the privacy paradox” [51]. Even people who express the highest degree of concern sometimes knowingly disclose personal information online [52, 53, cf. 54], and even those who are technically skilled or confident in their ability to protect their own privacy often fail to protect their privacy [55, 56]. Most users are simply unwilling to invest the time and energy required to assure the protection of their personal information, and, generally speaking, people’s privacy concerns are easily overridden by the various ways in which they benefit by disclosing information [28, 57–59, cf. 60]. For example, simple benefits such as monetary discounts or rewards tend to increase disclosure [61–64]. Privacy concerns are also overridden by perceived control; because people believe that they are powerless against data collection, they often fail to take steps to protect their privacy [65, cf. 66].

The risk/reward model may be only partially relevant to the privacy paradox, however. Because of the rapid and highly interactive way in which users interact with computers and mobile devices, they often don’t have time to make decisions about the information they are asked to disclose [67]. They are simply reacting mindlessly to queries, clicking on buttons or boxes, or pressing the Enter key without giving much thought to what they are doing [68].

### 2.5 Predictors of privacy behavior

Age and personality traits can be predictive of disclosure. Younger adults are more likely to disclose personal information than older adults [69, cf. 70]; more extroverted people and those who report low self-control are more likely to disclose intimate information online [71]; and those who rank higher in openness, lower in conscientiousness, and lower in agreeableness are more likely to disclose more information online [72]. Privacy awareness and confidence in one’s own ability to mitigate privacy concerns can predict privacy decisions [56]. Situational factors also significantly impact people’s privacy decisions. For example, the tendency to disclose is higher in large rooms than in smaller ones [73], and familiar environments where people are likely to feel a greater sense of protection may lead to higher trust and higher disclosure [74]. Disclosure is also higher when requests for information are indirect, rather than direct, and when website interfaces are unprofessional, rather than neutral or professional [75]. People are also more likely to pay for more privacy (for example, by shopping at a different website) when a privacy warning is too salient [76]. The perceived sensitivity of the information requested is another predictor of privacy behavior [77], and because different types of information, such as location, health status, and browsing history, are valued differently by different people, one cannot expect privacy behaviors to be consistent across situations [78]. After being told that other people have revealed certain types of information, people are more likely to reveal similar information themselves [79], and a similar phenomenon has even been observed when people interact with an avatar; people reveal more information to an avatar after it has shared information about itself [80]. Self-disclosure activates the brain’s reward system, perhaps demonstrating its intrinsic value [81].

### 2.6 Methods for influencing privacy concerns and behavior

Privacy concerns and privacy-protective behavior can each be impacted in various ways. Although rewards can increase disclosure, some studies have demonstrated that the offer of a reward for disclosing private information can increase privacy concerns [82], especially when the sensitivity of the information requested is high [83]. Including a privacy policy on a website has been shown to increase trust, which is associated with a decrease in disclosure concerns and increased willingness to disclose personal information [84–86]. When a privacy policy is presented as a formal, legalistic agreement, however, trust can deteriorate [87]. Some studies highlight the significance of certain policy features; to increase privacy-protective behaviors, information relevant to privacy decision making must be salient, easily accessible, complete, and threatening [76, 82, 88, cf. 89].

Timing is also important. When people are reminded about privacy at the moment they must make a decision, previously dormant privacy concerns might be awakened, leading to more privacy-protective behavior [90, 91]. This might occur because when users cannot easily bring risks to mind, they mistakenly perceive risk to be low [92].

Certain design features can be used to “push” users to make certain privacy decisions. Nudges–subtle attempts to influence people’s decisions without force [93]–have been used to improve privacy outcomes without limiting users’ choices [94]. For example, presentation nudges are used to provide necessary contextual clues to reduce the user’s cognitive load and convey the appropriate level of risk in order to mitigate biases and heuristics relevant to privacy decision-making [94]. Nudges can draw users’ attention to privacy links and decrease the posting of personal information to public audiences online [95, 96, cf. 97], and nudges that inform users about how they can mitigate privacy risks are more effective at increasing privacy-protective behavior than nudges that rely purely on fear [98]. Priming–exposure to relevant stimuli that influences the response to subsequent stimuli, regardless of awareness [99]–has also been used to deter the disclosure of personal information [100]. Framing–the way an outcome or situation is presented to an audience [101]–is another feature that influences people’s privacy-related decisions [102, 103].

These days, it is increasingly common to see a privacy-related pop-up box–or “cookie consent banner”–whenever one visits a new website. Sometimes the banner informs users that by proceeding onto the website, they are allowing the website owner or its agents to install a variety of unspecified tracking software on their computers; there is no way to opt out of this option. This is referred to as a browsewrap agreement [104]. At other times, the banner lets people click buttons that allow them to limit the tracking to what the website owner considers to be “essential” (which is generally undefined); this is referred to as a clickwrap or click-through agreement. Although clickwrap agreements might have been meant to increase user awareness of privacy threats [105], these banners are often structured in a way that encourages people to surrender their privacy [106]. For example, buttons reading “Join” or “I agree” are often visually more prominent than alternative buttons [68]–a dark pattern that has been shown to increase user acceptance [107]. Whether clickwrap agreements affect people’s tendencies to disclose sensitive information is unknown.

In the present study, we sought to determine the extent to which click requirements and privacy warnings would cause people to withhold sensitive personal information. It employed a randomized, controlled, 2 × 2 factorial design with a diverse sample of participants. Our design also included a control group–people who were not shown privacy warnings and who were not required to click on a clickwrap agreement.

## 3. Methods

The federally registered Institutional Review Board (IRB) of the sponsoring institution (American Institute for Behavioral Research and Technology) approved this study with exempt status under HHS rules because (a) the anonymity of participants was preserved and (b) the risk to participants was minimal. The IRB also exempted this study from informed consent requirements (relevant HHS Federal Regulations: 45 CFR 46.101(b)(2), 45 CFR 46.116(d), 45 CFR 46.117(c)(2), and 45 CFR 46.111). The IRB is registered with OHRP under number IRB00009303, and the Federalwide Assurance number for the IRB is FWA00021545.

### 3.1 Participants

Our participants were recruited from Amazon’s Mechanical Turk (MTurk) website, which has been used by social scientists in a variety of research since 2005 [108]. In all, 1,622 people were randomly assigned to each of 5 groups. Because people take different amounts of time to complete their sessions, we ended up with unequal numbers of people in each group: 306 in Group 1, 307 in Group 2, 327 in Group 3, 314 in Group 4, and 368 in Group 5. After separating the participants in each group who either dropped out of the study after providing demographic information (by closing the browser tab) or who quit the study after seeing the questionnaire (by clicking on an “end session” button), we were left with 304 in Group 1 (2 drops or quits), 304 in Group 2 (3 drops or quits), 304 in Group 3 (23 drops or quits), 305 in Group 4 (9 drops or quits), and 333 in Group 5 (35 drops or quits). Finally, to get an even number of people in each of the groups, we used SPSS’s “Random sample of cases” feature to select random samples of 300 people from each group. Our analysis therefore focused on five groups with 300 people in each. We were also able to preserve some information about 72 other people who dropped out of the study before completing it. The dropouts proved to be important in our analysis of the data (see below).

Participants ranged in age from 18 to 82 (*M* = 32.63 [*SD* = 10.78]). 853 (56.9%) identified themselves as male, 642 (42.8%) as female, and 4 (0.3%) as Other; gender was not reported for 1 (0.1%) of our participants. 1,215 (81.0%) of our participants were from the US, 259 (17.3%) were from India, and 26 (1.7%) were from 18 other countries. 949 (63.3%) of our participants identified themselves as White and 551 (36.7%) as Non-White in the following categories: 354 (23.6%) as Asian, 91 (6.1%) as Black, 49 (3.3%) as Hispanic, 20 (1.3%) as American Indian, and 37 (2.5%) as Other.

Level of education also varied over a wide range: no high school degree: 6 (0.4%); completed high school: 409 (27.3%); associate’s or 2-year degree: 244 (16.3%); bachelor’s degree: 642 (42.8%); master’s degree: 178 (11.9%); doctoral degree: 21 (1.4%). On a 10-point scale, where 10 was the highest degree of fluency, participants rated their English fluency as high (*M* = 9.63 [0.86]).

The 72 dropouts were similar to the 1,500 participants who completed the experiment in age [*t*(1570) = -1.627, *p =* 0.104, *d* = 0.19], gender (*z* = 0.68, *p =* 0.497), education (*z* = 0.66, *p =* 0.509), and race/ethnicity (*z* = 0.53, *p* = 0.603).

### 3.2 Procedure

Participants were directed from the MTurk site to our own web page where they were first asked basic demographic questions. In order to protect the identities of our participants (a requirement of the exempt status granted by the institutional review board of our sponsoring institution), participants were not asked for their full names or email addresses.

**Fig 1:**
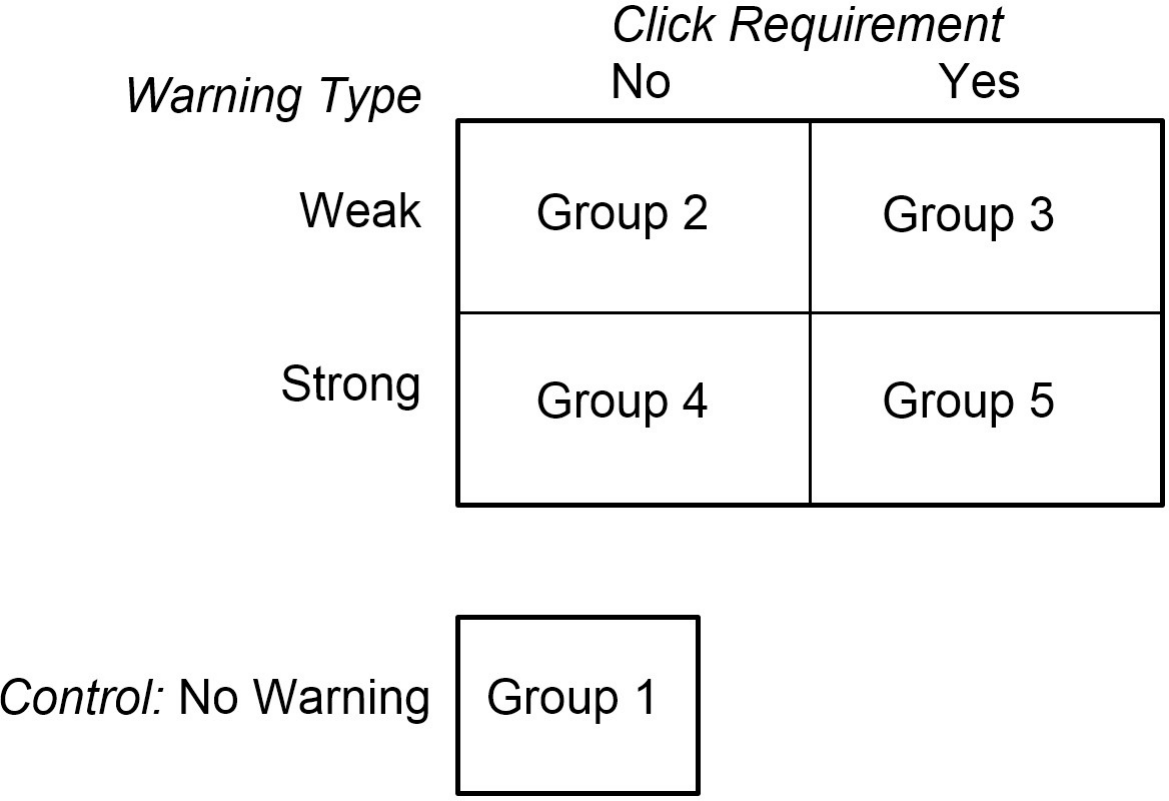
Experimental design. Participants in the control group (Group 1) were not shown a warning above the questionnaire. Participants in the other four groups were presented two levels of warning and two click conditions (click or no click required) in a 2 × 2 factorial design as shown in the figure.

They were then instructed to complete a 25-item survey in which they were asked to indicate whether they had engaged in a number of illegal, immoral, or socially controversial activities within the past three years–activities such as driving recklessly, watching pornography, smoking marijuana, harming oneself, cursing God, and so on. Participants could respond by clicking “Yes,” “No,” “Maybe,” “Can’t remember,” or “Click here to end your session.” Participants could also terminate their session by closing their browser tab. The number of possible transgressions participants admitted to committing (by clicking “Yes” to items in our survey) served as our dependent variable. The 25 items on the questionnaire were selected from among 100 items we had studied in pilot procedures. We chose the 25 items that were most impacted by our warnings (indicated by the difference between the number of admissions in Groups 1 and 5, see Fig 1). After completing the questionnaire, participants were taken to a debriefing page and thanked for their participation.

Participants were randomly assigned to one of five different groups in which warnings of various sorts were displayed above the questionnaire (see S1 to S5 Figs to view the screens). Warnings stated how data might be used, and in some groups participants were required to click a link to confirm they had understood the warning they were shown. In the control condition, no warning accompanied the questionnaire. In the other four groups, two independent variables were varied in a between-subjects, 2 × 2 factorial design: strength of warning and click requirement (Fig 1).

The weak warning included standard internet language telling the participants that they must comply with a Terms of Service (TOS) agreement: “Please note: By using this website you agree to our Terms of Service” [109] (see S2 and S3 Figs). The strong warning included a brief paragraph reminding participants that their answers and IP addresses were being recorded and that their information might be shared with others, as follows (see S4 and S5 Figs):

**PLEASE READ:** By using this website you agree to our Terms of Service [109]. Specifically, you give us your consent to record and store your survey answer along with identifying information such as your IP address, as well as to share this information as required or permitted by law with authorized individuals, companies, organizations, or government agencies.

In both warning conditions, participants could click a link to access a lengthy, detailed TOS agreement that contained two links to a lengthy, detailed privacy policy [110] (3,591 words in total). Both documents were modeled after corresponding Google documents. The number of people who clicked these links and the total time they kept these documents open were recorded. Participants in the click groups were required to click on the phrase “Please click here” to acknowledge that they had read and agreed to the TOS.

## 4. Results

### 4.1 Analysis of variance

**Fig 2:**
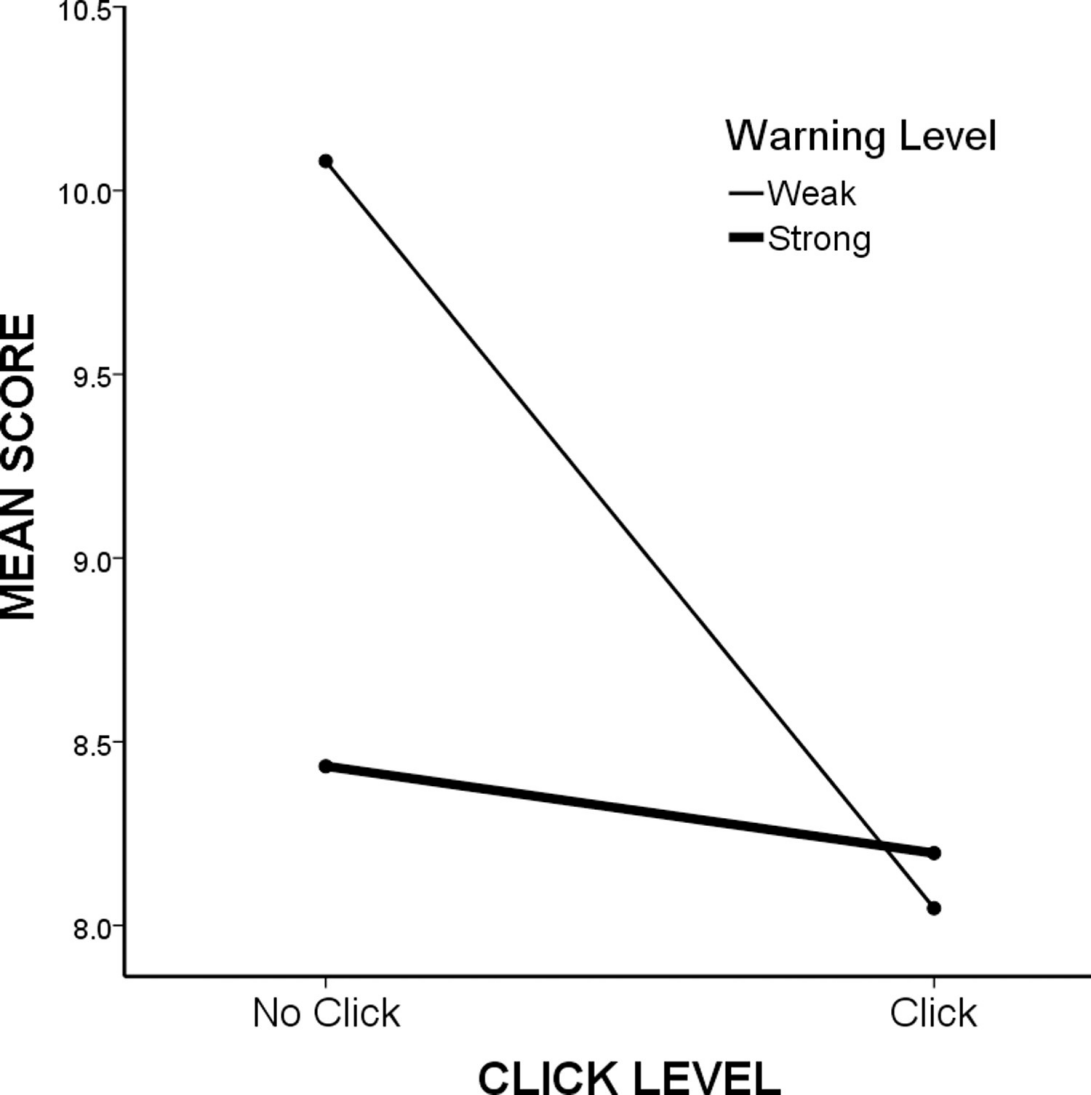
Graphical results of two-way ANOVA. It shows click level (no click or click) versus mean scores (mean number of “yes” responses). The thin line shows data for the weak warning condition, and the thick line shows data for the strong warning condition.

A two-way ANOVA of results in the four experimental groups (2, 3, 4, and 5) revealed main effects for both of our independent variables: warning strength (*M*_strong_ = 8.32 [5.31], *M*_weak_ = 9.06 [5.35], *p* < 0.05) and click requirement (*M*_click_ = 8.12 [5.32], *M*_non-click_ = 9.26 [5.30], *p <* 0.001). We also found a statistically significant interaction between these variables: *F*(1, 1196) = 8.674, *p* < 0.01 (Fig 2).

### 4.2 Control group versus experimental groups

We also found a significant difference between the mean score of the control group (Group 1, no warnings) and the mean score of the four experimental groups combined (Groups 2, 3, 4, and 5) (*M*_*1*_ = 10.80 [5.13], *M*_*2-5*_ = 8.69 [5.34], *t*(1,498) = 6.16, *p* < 0.001, *d* = 0.40). Pairwise comparisons between the mean score of the control group (Group 1) and the mean scores of three of the four experimental groups (Groups 3, 4, and 5) also produced significant differences (*M*_3_ = 8.05 [0.31], *t*(598) = 6.46, *p* < 0.001, *d* = 0.76; *M*_4_ = 8.43 [5.27], *t*(598) = 5.57, *p* < 0.001, *d* = 0.46; *M*_5_ = 8.20 [5.35], *t*(598) = 6.08, *p* < 0.001, *d* = 0.50). It is notable that the difference in the mean scores between the control group and Group 2 –people receiving the internet’s common TOS warning with no click requirement–was not significant (*M*_2_ = 10.08 [5.21]), *t*(598) = 1.70, *p* = 0.09, *d* = 0.14). Where G signifies Group, we can summarize this pattern of results as follows:

G1=G2<G3=G4=G5

This pattern shows that when we looked at the amount of sensitive personal information people disclosed, either a strong warning or a click requirement suppressed disclosure significantly. Overall, participants provided 32.8% more information when they had no privacy warning (Group 1) than when they had a click requirement (Groups 3 and 5 combined, *M* = 8.13[5.32]), and participants provided 24.3% more information when they had no privacy warning (Group 1) than when they had either a click requirement or a warning (Groups 2, 3, 4, and 5 combined, *M* = 8.69[5.34]).

### 4.3 Demographic differences

We found a marked difference between disclosures by US participants (Groups 2, 3, 4, and 5 combined, *N* = 920, *M* = 9.93 [5.01]) and disclosures by participants from India (Groups 2, 3, 4, and 5 combined, *N* = 257, *M* = 4.31 [4.07]) (see Discussion). We also found significant differences in disclosures by gender (*M*_male_ = 9.80 [5.22], *M*_female_ = 8.21 [5.42], *t*(1493) = 5.76, *p* < 0.001), *d* = 0.30, race/ethnicity (*M*_White_ = 10.34 [4.95], *M*_Black_ = 8.87 [4.93], *M*_Hispanic_ = 10.61 [4.92], *M*_Asian_ = 6.00 [5.16], *M*_AmIndian_ = 7.95 [6.15], *M*_Other_ = 6.51 [5.72], *F*(5, 1494) = 41.42, *p* < 0.001), and education (*M*_none_ = 7.83 [5.19], *M*_highschool_ = 10.72 [5.03], *M*_associates_ = 9.93 [5.43], *M*_bachelors_ = 8.44 [5.34], *M*_masters_ = 6.88 [4.88], *M*_doctorate_ = 8.14 [5.48], *F*(5, 1494) = 17.79, *p* < 0.001), as well as an effect for age (*r* = -.22, *p* < 0.001).

### 4.4 Impact and characteristics of dropouts

**Fig 3:**
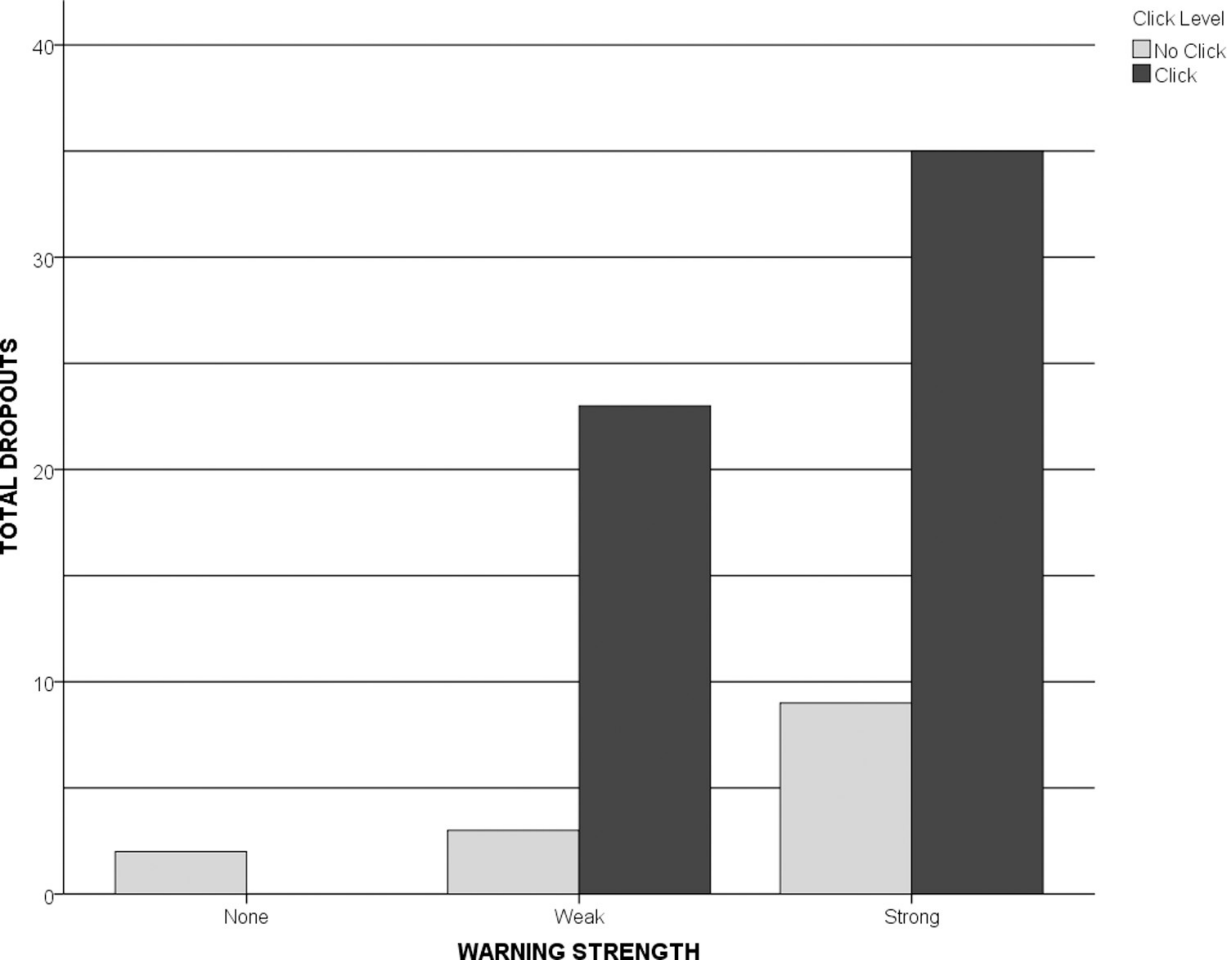
Pattern of dropouts. Written warnings alone drove only a few people away from the study. An added click requirement increased the total number of dropouts substantially (black bars).

The power of the click requirement is revealed further when one looks at the pattern according to which people either dropped out of the experiment before completing it by closing their browser tab or by clicking a button we provided which read, “If you have decided not to complete the survey, please click here to end your session” (Fig 3, Table 1). (Henceforward, we will refer to both categories combined under one label: “dropouts.”).

**Table 1:** Comparison of dropouts by group number.

Group No.	Total Dropouts	Attrition Rate	Comparison Group	z-score	*p* value
**Group 1**	2	0.007	Group 2	0.04	0.653
**Group 2**	3	0.010	Group 4	1.71	0.087
**Group 3**	23	0.070	Group 5	1.18	0.238
**Group 4**	9	0.029	Group 3	2.42	< 0.05
**Group 5**	35	0.095	−	−	−
**Groups 2 thru 5**	70	0.053	Group 1	3.57	< 0.001

The attrition rate in the control group (0.007) was significantly lower than the attrition rate in the experimental groups combined (0.053, z = 3.57, *p* < 0.001) (Table 1). We also found a significant difference in attrition rates across the five groups individually (*χ*^*2*^[4, *N* = 1,622] = 48.35, *p* < 0.001). Pairwise comparisons of attrition rates revealed another interesting pattern:

G1=G2=G4<G3=G5

In other words, the click requirement (present in Groups 3 and 5 only) affected attrition significantly.

### 4.5 Terms of service warning

Of the 1,200 people who were prompted to view the TOS agreement, only 88 (7.3%) did so, and only 17 (19.3% of those who viewed the TOS agreement) clicked through to the privacy policy. The average amount of time these people kept these documents open was 22.9 seconds, roughly enough time to read 91 words (2.5% of the total) [111].

## 5. Conclusions

Our results support four conclusions: (1) The commonly-used TOS warning has no deterrent effect and is functionally the same as no warning at all. (2) A strong, more explicit, warning has some deterrent effect. (3) A click requirement increases the effectiveness of both weak and strong warnings, and it can also cause people to close a web page. (4) Given that most, or perhaps nearly all, internet users are exposed either to no warnings regarding the possible fate of the information they are providing (our Group 1), or are exposed at most to some mention of a Terms of Service agreement (our Group 2), our results suggest that internet users may currently be disclosing at least 32.8% (our Groups 3 and 5) more personal information than they otherwise would if they were more effectively warned about the risks involved.

## 6. Discussion

### 6.1 Insights on attrition

Although we detected only 72 people who left our study before completing it, these people are in some respects the most interesting and revealing in the study. They are interesting from the perspective of experimental design because most studies never track such people. In our early pilot experiments, neither did we, and that sometimes gave us misleading results. When we had a 100-item questionnaire, we sometimes found no effects, presumably because of large attrition rates. With a short questionnaire, we found clear effects among the people who completed the survey, and we also found a clear pattern of attrition associated with the different groups.

The dropout pattern is also interesting in what it might be telling us about how the internet is segmenting societies worldwide. In Dave Egger’s 2013 book, The Circle (subsequently made into a movie starring Emma Watson and Tom Hanks), surveillance by a Google-like company has become so pervasive and extreme that some people are going to great lengths to go “off-grid” [112]. The main character, Mae Holland–a rising star at the company–loves the surveillance, but her ex-boyfriend does not. To escape the invasive electronics, he moves to a cabin in the woods, at which point Mae asks her huge cadre of online followers to find him. Minutes later, camera-carrying drones surround his home, at which point he jumps into his pickup and drives straight off a bridge to his death. In other words, he went off-grid by literally going off-grid.

Our dropouts might be giving us a glimpse of yet another aspect of a dark electronic future. They are still connected, but they apparently don’t like divulging sensitive personal information. Completely absent from our study is a much larger group of people who are already disconnected–who have quit social media platforms or perhaps never even got hooked. In age, race, gender, and education, our dropouts looked just like the people who completed our study, but we suspect they differed markedly in personality characteristics. Did our dropouts have higher perceived self-efficacy than our finishers [113–115]? Were our finishers more extroverted and open, less conscientious, or perhaps even more exhibitionistic [72, 116, 117] than our dropouts? The billions of people who post messages, photos, and videos of themselves on social media platforms every day hardly seem shy, although some might be sharing their lives online as a response to social pressure [118–120].

The internet might be dividing the world’s population into two distinct groups: people we might call “LoudMouths,” who compete each day for attention and followers, and people we might call “ZipMouths,” who are largely absent from the space that has become increasingly dominant in our lives: Cyberspace. With more and more social science research moving online [121, 122, cf. 123], are important studies drawing erroneous conclusions because of how the internet is segmenting societies? Are we basing our research conclusions on samples that exclude certain personality types? And with major news outlets routinely basing news stories on social media trends [124] and many people turning to social media to get the latest news [125, 126, cf. 127], are Zipmouths losing their ability to influence social policy–perhaps even to influence the outcomes of elections?

What if this trend continues? Although it is clearly in the interest of online entities to extract as much personal data from users as possible, authorities are gradually forcing web hosts to inform users about the risks associated with using their web pages. We see this trend in the increasing number of pop-ups warning us about cookies and other invasions of privacy, some of which now include a click requirement [128]. This practice might cause some people to close a web page and others to divulge less information. Over time, however, such practices will also drive more people off-grid–and, potentially, outside the bounds of a functioning society.

### 6.2 The power of the click requirement

Warnings–along, of course, with all the fake news, trolling, and bullying–might drive some people off the internet because of their official, legalistic appearance and content. They create the impression that the user is entering into a binding legal contract. A growing body of law in the US suggests, however, that the appearance of a TOS warning alone is not legally binding, but when a user clicks his or her assent to such a warning, courts have ruled that the agreement is binding [27, 39, 104]. The legalistic language in our strong warning might have been essential to its impact [see 87]. That issue should be explored in future research.

Our findings on dropouts also suggest that in studies in which attrition can have a systematic effect on study outcomes, it is essential that attrition be closely tracked. Recall that in some of our pilot experiments (when we used a 100-item questionnaire), we sometimes failed to find effects, almost certainly because we failed to track dropouts.

How effective various types of warnings are in discouraging personal disclosures online is a complex issue. It depends not only on the nature of the warning but also on the value users perceive in divulging such information. In the highly exhibitionistic environments of Facebook, Reddit, and Instagram, photos and disclosures–the more extreme, the better–bring comments, likes, and followers, all of which increase people’s social capital, thus increasing their tendency to use social media and disclose more online [117, 129–131]. Disclosing personal information also allows platforms like Google and Facebook to target ads more precisely. For some people, those ads turn the internet into their personal shopper; for others, they are reminders of privacy lost. When we contemplate the power of warnings and click requirements, we also need to think about the rewards associated with the behaviors we are trying to suppress [132]. In many cases and for many people, attempts to suppress disclosures are little more than annoyances [32].

Why a click requirement had such a large impact in our experiment is unclear, but we suspect that this is an attentional phenomena. A click box is a graphical element that draws attention, especially when a click is required in order for a user to proceed. Graphical elements that draw attention on a computer screen have been shown to have a greater impact on user behavior than more subtle graphical elements [133, 134, cf. 135, 136], and that finding is consistent with a long history of research on attention in various contexts [2, 137, 138]. Because required clicks near a warning message also suggest legal liability (which is, as we noted, supported by emerging case law), it is also possible that users who encounter a click requirement are more likely to fear the associated warnings. Our Groups 3 (click requirement with weak warning) and 5 (click requirement with strong warning) begin to shed some light on such issues, but further research, including eye-tracking studies, must be conducted to learn precisely why the click requirement is so powerful.

### 6.3 Limitations and concerns

The validity of the present study is limited by its sample–a group of people recruited from Amazon’s MTurk subject pool. Most were from the US (81.0%), but a sizeable group was from India (17.3%). Further research on warnings and click requirements should reach out to different samples, especially in cultures and countries outside the US [139]. As noted earlier, we found significant and, sometimes, surprisingly large differences in disclosure rates by different demographic groups. Participants from the US, for example, disclosed more than twice as many sensitive activities (M = 9.93 [5.01]) as participants from India did (M = 4.31 [4.07]). That difference could be explained by cultural differences that have been studied by anthropologists and other social scientists [140, 141]. Our study was not structured in a way, however, that allows for meaningful comparisons to be made between different cultures.

Future research on factors affecting online disclosures should also look specifically at (a) types of disclosure that are actually common online, such as information about people’s personal lives, along with photos and videos, and (b) how context and environment impact disclosures. Disclosure is the norm, for example, on one’s Facebook or Instagram pages, but it often occurs without people’s knowledge when they use Google’s search engine or Gmail. Warnings and click requirements will almost certainly have to take on very different forms to be effective in the wide range of environments that people now inhabit online, and privacy-promoting techniques that work well with one demographic group might work poorly with another.

Disclosures are also now the norm when people are interacting with personal assistants such as Apple’s Siri, Microsoft’s Cortana, Amazon’s Alexa, and the Google Assistant (standard on Android devices). It is not at all clear how, query by query and device by device, we can meaningfully warn people about the fact that they are disclosing personal information, possibly to their detriment. An increasing body of evidence also indicates that these and other personal assistants constantly record whatever they are hearing [142–144]. Again, how can we meaningfully warn people about invisible surveillance that never stops?

The growing internet of things is rapidly complicating the disclosure problem. In 2014, for example, Google bought Nest Labs [145]. Several years later, it was revealed that Google had installed microphones into the Nest Guard alarm system without disclosing this to users [146]. When the company was called out, it could hardly deny the existence of the microphones, but it claimed it had not yet activated them (then why install them?) [147].

There is good news and bad news here. The good news is that click requirements seem to be surprisingly powerful in discouraging people from disclosing personal information. The bad news is that corporate surveillance is so pervasive and aggressive and so thoroughly embedded into the online environment that no attempts to discourage personal disclosure are likely to make much difference. We join with other scholars and scientists in calling upon our leaders to make the surveillance business model–a fundamentally deceptive model that was invented by Google and that is now being imitated by thousands of businesses worldwide [148]–illegal [148–152].

## Supporting information

S1 Fig Group 1 screen (partial view). (TIF)

S2 Fig Group 2 screen (partial view). (TIF)

S3 Fig Group 3 screen (partial view). (TIF)

S4 Fig Group 4 screen (partial view). (TIF)

S5 Fig Group 5 screen (partial view). (TIF)
