# Supplementary figures and images for: The surprising power of a click requirement: How click requirements and warnings affect users’ willingness to disclose personal information
Source: PLoS One. 2022 Feb 18;17(2):e0263097. doi: 10.1371/journal.pone.0263097 (PMC8856545; doi:10.1371/journal.pone.0263097)

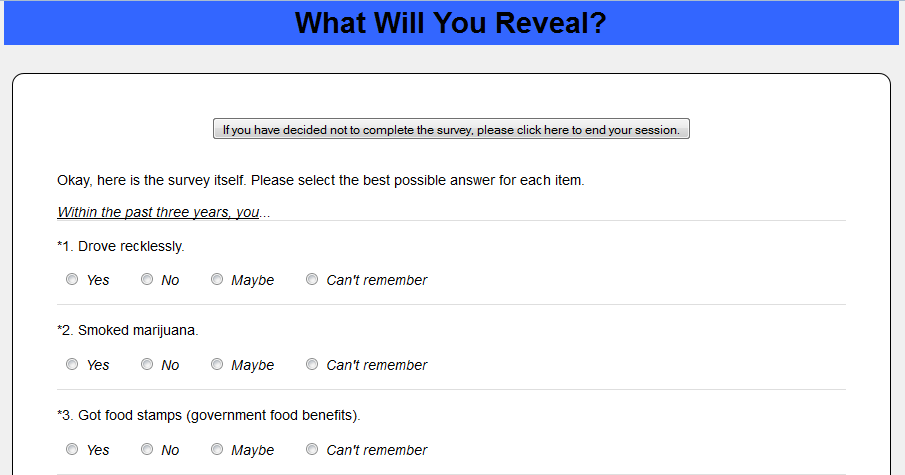

Supplement: S1 Fig — (TIF) [file pone.0263097.s001.tif]

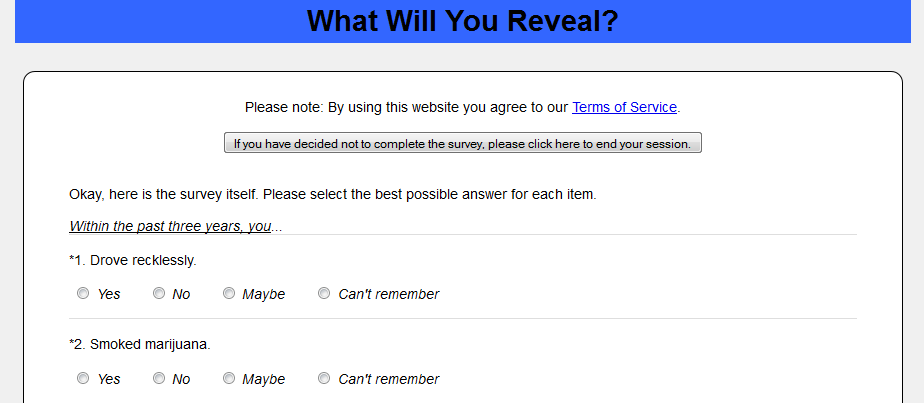

Supplement: S2 Fig — (TIF) [file pone.0263097.s002.tif]

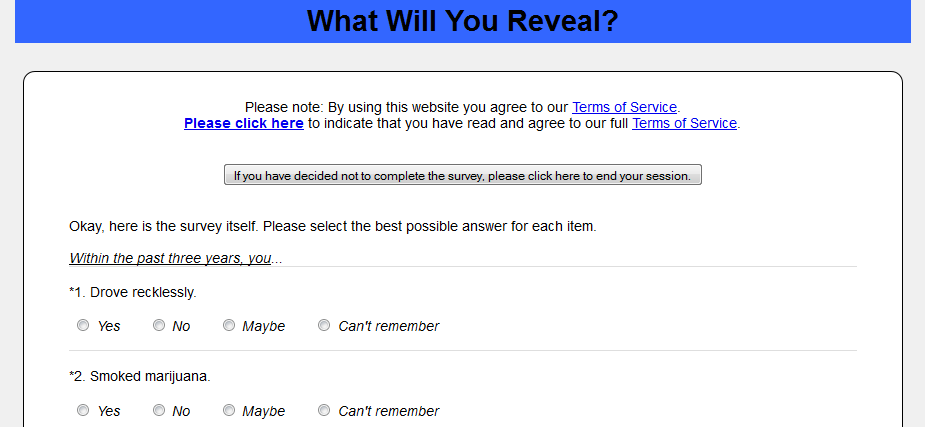

Supplement: S3 Fig — (TIF) [file pone.0263097.s003.tif]

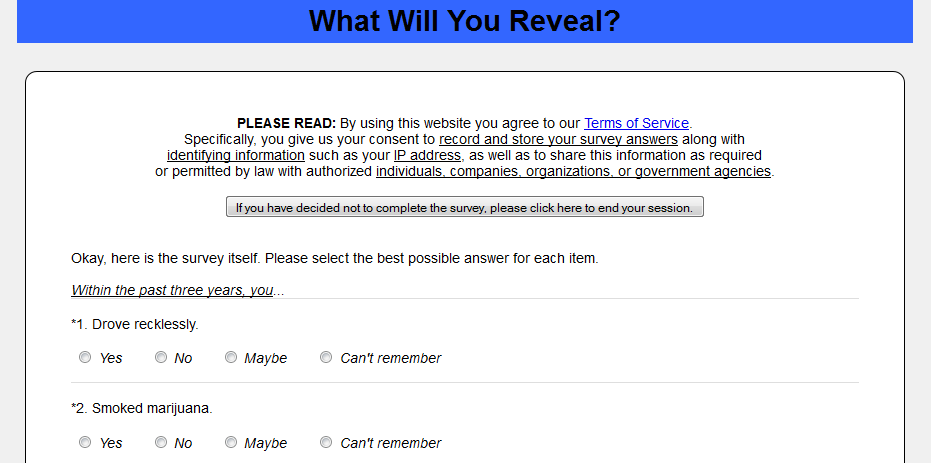

Supplement: S4 Fig — (TIF) [file pone.0263097.s004.tif]

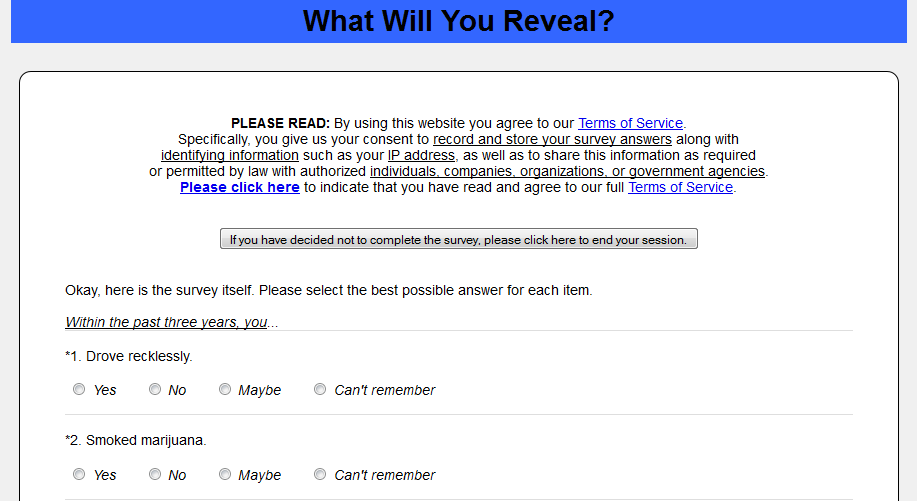

Supplement: S5 Fig — (TIF) [file pone.0263097.s005.tif]
